# Long-term follow-up after surgical management of laryngeal malignant pleomorphic sarcoma ‒ a case report

**DOI:** 10.1016/j.bjorl.2023.05.003

**Published:** 2023-05-19

**Authors:** Giorgio Bandiera, Edoardo Covelli, Haitham H. Elfarargy, Evelina Rogges, Maurizio Barbara, Luigi Sabino, Chiara Filippi

**Affiliations:** a Sapienza University, Sant′ Andrea University Hospital, Mental Health, and Sense Organs (NEMOS), Department of Neuroscience, Rome, Italy; b Kafrelsheikh University, Otorhinolaryngology Department, Kafrelsheikh, Egypt; c Sapienza University, Sant′ Andrea University Hospital, Department of Clinical and Molecular Medicine, Rome, Italy

## Introduction

Sarcomas of the head and neck are rare malignant tumors accounting for less than 1% of malignant tumors in this region and less than 10% of all soft tissue sarcomas. Head and neck sarcomas are mainly present in the neck, face, forehead, and sinuses. While laryngeal sarcoma is rare, comprising less than 1% of all laryngeal tumors. These neoplasms display a diverse array of histology and a broad spectrum of clinical activity ranging from relatively slow-growing lesions to aggressive locally and regionally destructive lesions with the potential for systemic metastasis. In addition, these neoplasms' anatomic and pathophysiologic heterogeneity demands variable and multifactorial considerations for effective management.^1^, ^2^, ^3^

Sarcoma of the head and neck commonly presents as a painless submucosal or subcutaneous mass of uncertain duration. The early symptoms of laryngeal sarcoma result from mechanical interference with function and depend on the size and rate of the tumor growth. They are, as a rule, insidious in onset and progression. Hoarseness is usually the first symptom noted. Stridor and dyspnea follow over time unless the tumor is removed at its early stages. Dysphagia is not likely to be a prominent symptom, especially at the early stage, until it becomes large enough and protrudes into the hypopharynx.^4^, ^5^

Neither the symptoms nor the physical findings are sufficiently characteristic in cases of laryngeal sarcoma to permit a clinical diagnosis. An accurate diagnosis depends on a careful biopsy by a laryngoscope under general anesthesia. It is essential to get the core of tissue and not only the superficial tumor. A complete study by an experienced pathologist and extensive immunohistochemistry tests to distinguish various types of soft tissue sarcomas are usually required.^6^

More than 50% of laryngeal sarcomas are fibrosarcoma and chondrosarcoma. Osteosarcoma, liposarcoma, undifferentiated pleomorphic sarcoma, synovial sarcoma, and rhabdomyosarcoma are rare. Laryngeal leiomyosarcoma is extremely rare.^6^

This study aimed to shed light on a case presented with undifferentiated high-grade pleomorphic transglottic sarcoma who underwent only laryngectomy. The 5-year postoperative follow-up did not reveal any recurrence or deterioration.

## Case presentation

**Figure 1:**
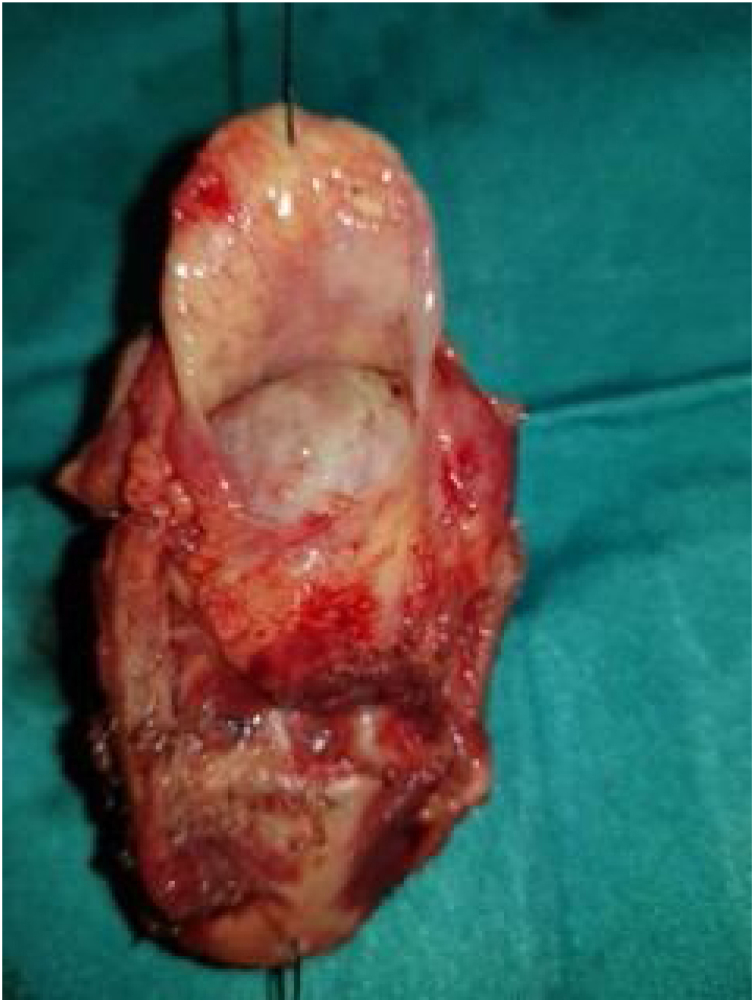
Preoperative endoscopic image of the mass in the clinic showing a glottic exophytic mass encroaching the anterior commissure of both vocal folds in a horse-shoe shape manner.

**Figure 2:**
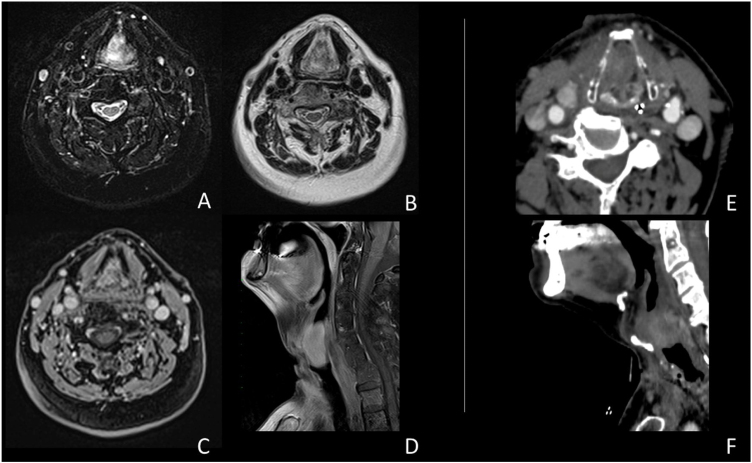
(A‒F) MRI study (A‒D); CT study (E‒F). The images document an expansive glottic lesion characterized by homogeneous enhancement after contrast injection in CT and MRI studies. In addition, obliteration of the glottic space is evident, with anterior dislocation of the thyroid cartilage shield without visible involvement of the paraglottic area and the cartilaginous structures.

**Figure 3:**
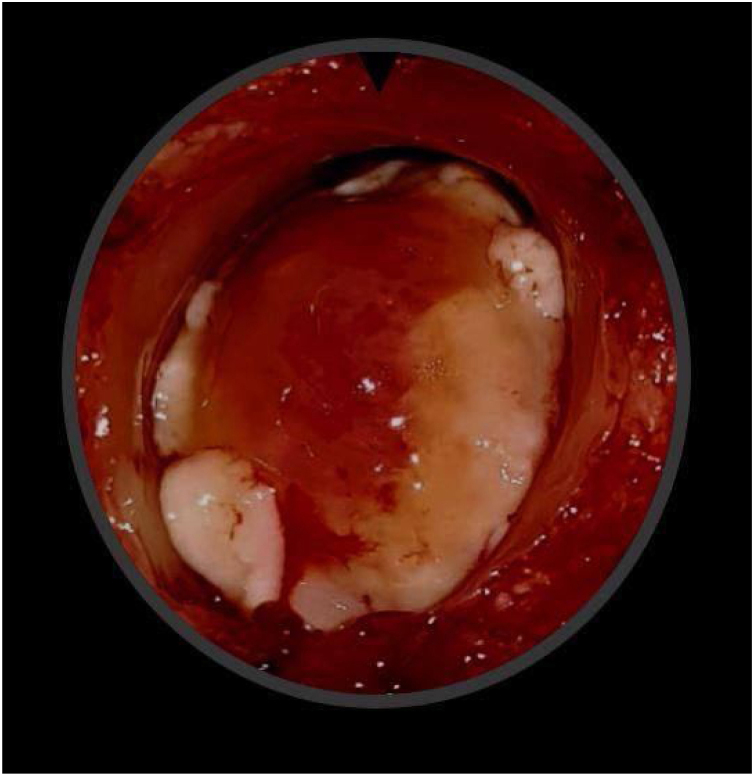
Intraoperative surgical specimen after total laryngectomy showing the transglottic mass occupying the laryngeal lumen without extralaryngeal extension.

**Figure 4:**
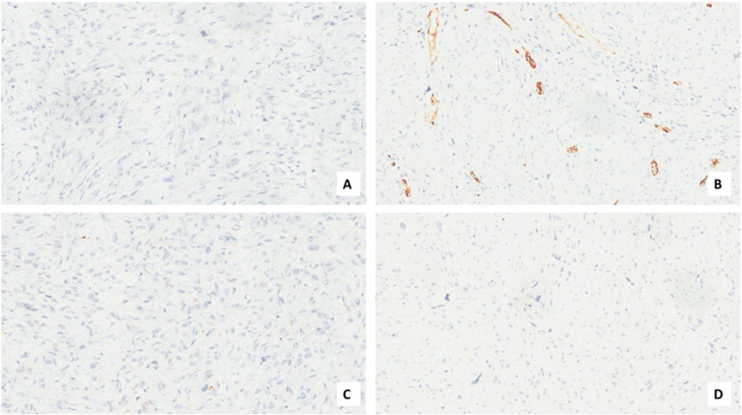
Histopathology of the larynx lesion (A–D). Fascicles and sheets of spindle cells are arranged in a storiform pattern. (A, Hematoxylin and Eosin 200×, upper insert 20×) expressing CD68 (B, 200×), p53 (C, 200×), with 32% of proliferation index (D, Ki-67, 200×).

**Figure 5:**
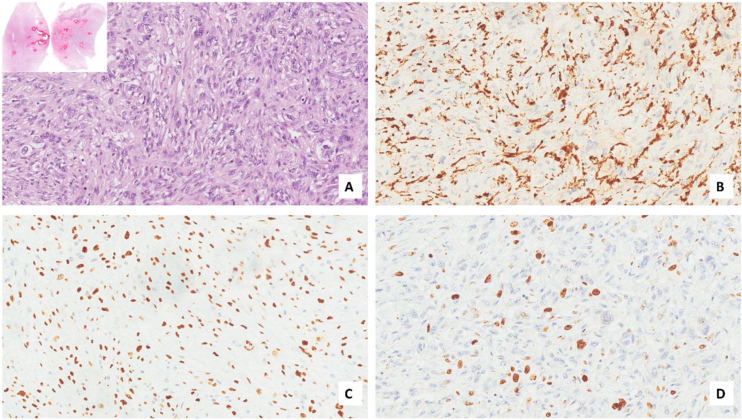
Histopathology of the larynx lesion (A–D). Neoplastic cells were negative for CKAE1/AE3 (A, 200×), CD34 (B, 200×), S100 (C, 200×), and Desmin (D, 200×).

A male patient, age 71 years, presented to the emergency department of our hospital with severe stridor and dyspnea, and the oxygen saturation in the air was 87%. In the previous three months, he had a gradual onset and progressive course of hoarseness of voice and dyspnea, especially on effort. Still, the condition deteriorated suddenly in the last week. He was a tobacco smoker 53 years ago, not diabetic or hypertensive, and there was no family history of a similar condition or malignancies. Endoscopic examination of the larynx in the emergency department revealed the presence of a glottic exophytic mass encroaching the anterior commissure of both vocal folds in a horse-shoe shape manner with bilateral fixed vocal folds, and the respiratory chink was very narrow (less than 1 cm) (Fig. 1). The patient underwent an urgent life-saving tracheostomy under general anesthesia. The operation also included microlaryngosurgery for tumor mapping and taking biopsies from the mass and the suspicious areas of the larynx. The histopathological examination of the mass showed an undifferentiated stromal malignancy with variable CD68 positivity, nuclear expression of p53 in 50% of the lesion, and the proliferation index assessed by Ki67 expression analysis was 55%. All findings suggested sarcoma of the larynx. According to these results, the patient underwent Computed Tomography (CT) neck with contrast and a gadolinium-enhanced Magnetic Resonance Imaging (MRI), which revealed the presence of a right glottic soft-tissue mass that extended anteriorly to involve the anterior commissure of both vocal folds and affected the anterior 2/3 of the left vocal fold obscuring the glottic area without infiltration of the thyroid cartilage. The adipose tissue of the anterior part of the right para-glottic space was infiltrated; in contrast, the right posterior para-glottic and left para-glottic spaces were not infiltrated. It extended superiorly, infiltrated the supraglottic area obscuring the ventricles of Morgagni, and reached the posterior aspect of the epiglottis with infiltration of both aryepiglottic folds. It also extended posteriorly, affecting the right posterior commissure without affecting the prevertebral muscle and facia. Moreover, the subglottic area was infiltrated without affecting the cricoid cartilage. There was no radiological evidence of cervical lymph node involvement (Fig. 2). Radiological workup included CT chest and brain, abdominal ultrasound, and Positron Emission Tomography (PET) scan excluded distant metastasis. All these results suggested the presence of a T_3_ N_0_ M_0_ laryngeal tumor. After the patient and his relatives' counselling and the complete preoperative evaluation, the patient underwent a total laryngectomy operation without neck dissection. The gross examination of the tumor specimen showed that it was a polypoid lesion with a gray-white cut surface (Fig. 3). The greatest dimension was 5.2 × 2.5 × 1.5 cm with a 0.5 cm base implant on the anterior commissure extending to the right vocal cord. The tumor completely obliterated the laryngeal space extending cranially and caudally to the glottal plane. No infiltration of the laryngeal walls was seen; on the contrary, the thyroid cartilage appeared atrophic due to the compression of the mass with hemorrhagic stripes and a gelatinous aspect. The histological examination showed fascicles and sheets of spindle cells in a storiform pattern. Numerous markedly atypical cells and some multinucleated giant cells were admixed with pleomorphic tumor cells. Chronic inflammation and abundant atypical mitosis (16 mitoses × 10 HPF), necrosis, and hemorrhagic areas were also present. The proliferative index, valued with Ki67, was 32%. The tumor cells showed focal expression of SMA and CD68 and negative expression for pan-CK, S-100, and Desmin. Moreover, 45% of neoplastic cells exhibit nuclear expression of P53. So, the histopathological diagnosis was Undifferentiated Pleomorphic Sarcoma (UPS) with giant cells (Figure 4, Figure 5). The resection margins were free. The patient was discharged from the hospital two weeks after the surgery and didn't receive postoperative radiotherapy. He underwent a restricted follow-up office endoscopic examination every three months. Five years after the surgery, he was generally fit without any signs or symptoms of tumor recurrence. This study followed the local institutional ethical standards and the Declaration of Helsinki. We obtained the patient's signed informed consent to involve his data in our research.

## Discussion

Undifferentiated High-grade malignant Pleomorphic Sarcoma of the larynx (UHPS) is also called Malignant Fibrous Histiocytoma (MFH). It is a rare histological type of laryngeal sarcomas. 43 cases of laryngeal pleomorphic sarcoma of the larynx have been described in literature since 1972.^3^ In men; laryngeal pleomorphic sarcoma is typically found in the glottic area. In contrast, in women in the subglottic region.^7^ Laryngeal pleomorphic sarcoma's etiopathology is still unclear, but recently, a relationship has been found between radiation exposure and the onset of the tumor.^8^ Our patient denied a history of treatment with or radiation exposure and had no other risk factors, indicating that MFH's etiology requires further investigation. This type of cancer doesn't show a great tendency to local infiltration, but it can grow rapidly, reducing the lumen of upper airways. The most common symptoms referred to by patients are hoarseness and dyspnoea.^9^

MFH is a high-grade malignant mesenchymal neoplasm characterized by pleomorphic tumor cells with variable morphology that can be fusiform or polypoid with mitosis proliferation which may be atypical and bizarre. The most common histological pattern is a mixture of pleomorphic and storiform areas. Usually, tumor cells are arranged haphazardly in sheets, and the stroma consists of delicate collagen fibrils between individual cells.^10^

For the diagnosis of this neoplasm, immunohistochemistry is a fundamental tool. It can also exclude other pleomorphic tumors such as sarcomatoid carcinoma, fibrosarcoma, myxofibrosarcoma, osteosarcoma, chondrosarcoma, and pleomorphic forms of liposarcoma and leiomyosarcoma. Diagnosis of UPS needs extensive sampling and evaluation of hematoxylin-eosin-stained sections, immunohistochemistry that shows a typical expression for Smooth Muscle Actin (SMA), a focal expression for cytokeratin, and stains for Desmin and Caldesmon are typically negative.^11^, ^12^

The mortality of laryngeal sarcoma is higher than other sarcomas occurring at the extremities. Because of its rarity, it is hard to define a clear five years overall survival, estimated at around 75%, and 5-year Disease-Free Survival (DFS) of nearly 50%.^13^

The most appropriate management of this rare tumor is the surgical excision with adjuvant radiation and chemotherapy for selected cases only. The type of surgery depends on the stage, and it can be a total laryngectomy, a partial laryngectomy, or a cordectomy. Also, it can involve neck dissection based on lymph node involvement.^14^

Radiotherapy (RT)'s role in treating head and neck sarcomas has evolved considerably over the past 30 years. Although, in many instances, sarcomas demonstrate considerable radio-resistance, RT remains an essential adjunct in treating soft tissue sarcoma to diminish the incidence of local recurrence.^15^ In our case, all the margins were free (negative), and the tumor was primary without extensive local destruction. So, we did not include postoperative RT in our management plan. This management plan was successful, as there was no recurrence in the following five years.

## Conclusions

Pleomorphic sarcoma of the larynx is a sporadic head and neck tumor and can be considered aggressive. Therefore, it’s essential to define a multidisciplinary therapeutic approach to manage this malignancy. If the margins are free, it can be managed surgically (total laryngectomy). A long-term follow-up is necessary to monitor any recurrences and improve disease-free survival.

## Authors’ contributions

Giorgio Bandiera: Designed the work; Edoardo Covelli: Acquired and analyzed data; Haitham H. Elfarargy: Acquired and analyzed data; Evelina Rogges: Acquired and analyzed data; drafted, revised, and approved the Maurizio Barbara: Drafted, revised, approved the manuscript; Luigi Sabino: Drafted, revised, approved the manuscript; Chiara Filippi: Agreed to be accountable for all aspects of the work.

## Funding

The authors have no budget or financial relationships to disclose.

## Conflicts of interest

The authors declare no conflicts of interest.

## References

[1] Cambruzzi E., Pedrini R., Vinícius C., Gava G., Pêgas K.L. (2020). Undifferentiated high-grade pleomorphic sarcoma of the larynx treated with a partial laryngectomy. Braz J Otorhinolaryngol..

[2] Van Damme J.P., Schmitz S., Machiels J.P., Galant C., Grégoire V., Lengelé B. (2010). Prognostic factors and assessment of staging systems for head and neck soft tissue sarcomas in adults. Eur J Surg Oncol..

[3] Testa D., Motta S., Marcuccio G., Paccone M., Rocca A., Ilardi G. (2016). Our experience in the treatment of Malignant Fibrous Hystiocytoma of the larynx: clinical diagnosis, therapeutic approach and review of literature. Open Med (Wars)..

[4] Tudor-Green B., Gomez R., Brennan P.A. (2017). Current update on the diagnosis and management of head and neck soft tissue sarcomas. J Oral Pathol Med..

[5] Aljariri A., Alsaleh A.R., Al-Enazi H.A., Haider H.A., Petkar M., Rahman W. (2021). Glottic malignant fibrous histiocytoma: a case report and literature review. Case Rep Oncol..

[6] Astl J., Holy R., Tuckova I., Belsan T., Pala M., Rotnagl J. (2021). Sarcomas of the larynx: one institution’s experience and treatment protocol analyses. Medicina (Kaunas)..

[7] Cao X., Liu J., Zheng Y., Li Q., Teng Y., Li Y. (2012). Simultaneous squamous cell carcinoma with primary malignant fibrous histiocytoma of the larynx: a case report. Mol Med Rep..

[8] Cahan W.G., Woodard H.Q., Higinbotham N.L., Stewart F.W., Coley B.L. (1998). Sarcoma arising in irradiated bone: report of eleven cases. 1948. Cancer..

[9] Karkos P.D., Dova S., Sotiriou S., Markou K., Kostopoulos I. (2016). Double primary malignant fibrous histiocytoma and squamous cell carcinoma of the larynx treated with laser laryngeal conservation surgery. Ecancermedicalscience..

[10] Engellau J., Anderson H., Rydholm A., Bauer H.C.F., Hall K.S., Gustafson P. (2004). Time dependence of prognostic factors for patients with soft tissue sarcoma: a Scandinavian Sarcoma Group Study of 338 malignant fibrous histiocytomas. Cancer..

[11] Coindre J.-M., Hostein I., Maire G., Derré J., Guillou L., Leroux A. (2004). Inflammatory malignant fibrous histiocytomas and dedifferentiated liposarcomas: histological review, genomic profile, and MDM2 and CDK4 status favour a single entity. J Pathol..

[12] Anghelina F.L., Ioniţă E., Chiuţu L., Mogoantă C.A., Ciolofan S., Iosif C. (2009). Malignant fibrous histiocytoma of larynx with giant cell: case report and histological - clinical considerations. Rom J Morphol Embryol..

[13] Andersen S., Mann H., Krarup-Hansen A., Lajer C.B., Grønhøj C. (2019). Patient and tumour characteristics of adult head and neck soft tissue sarcomas: a systematic review and meta-analysis. Sarcoma..

[14] Liu C.-Y., Wang M.-C., Li W.-Y., Chang S.-Y., Chu P.-Y. (2006). Sarcoma of the larynx: treatment results and literature review. J Chin Med Assoc..

[15] de Juan Ferré A., Álvarez Álvarez R., Casado Herráez A., Cruz Jurado J., Estival González A., Martín-Broto J. (2021). SEOM Clinical Guideline of management of soft-tissue sarcoma (2020). Clin Transl Oncol..

